# Predictive Value of the Proteomic Aging Clock and APOE Genotype for Incident Alzheimer’s Disease Risk

**DOI:** 10.1093/geroni/igaf122.2253

**Published:** 2025-12-31

**Authors:** Longjian Liu

**Affiliations:** Drexel University, Philadelphia, Pennsylvania, United States

## Abstract

**Aims:**

This study aimed to develop a novel proteomic aging clock (PAC) and evaluate its predictive value, along with APOE-ε4, for Alzheimer’s disease (AD) and AD-related dementias (AD/ADRD) risk in a cohort of adults ages 45–64 at baseline (1987–1989) with a median follow-up of 30 years.

**Methods:**

We analyzed data from 469 participants in the Atherosclerosis Risk in Communities (ARIC) Study, including plasma proteomic measurements (n = 460) and APOE genotyping. Aging-related proteomic features were identified using machine learning-based elastic net and Lasso regression. Cox proportional hazards models were used to retrospectively estimate hazard ratios (HRs) for AD/ADRD associated with PAC.

**Results:**

With a total follow-up of 9,366 person-years, the incidence rates (95% CI) of AD/ADRD per 1,000 person-years were 11.1 (8.8–13.9) in individuals without APOE-ε4, 15.9 (11.7–21.7) in those with one copy, and 23.7 (8.9–63.0) in those with two copies of APOE-ε4. Among the 460 candidate plasma proteomics, 43 were identified as key predictors. After adjusting for chronological age and other key covariates, the adjusted HRs (95% CI) for incident AD/ADRD by PAC quantiles (Q1 as the reference group) were: Q2: 1.63 (0.78–3.38), Q3: 1.13 (0.50–2.55), Q4: 2.48 (1.10–5.59), and Q5: 3.24 (1.43–7.34). African Americans had a significantly higher AD/ADRD risk than Whites (HR = 2.14, 95% CI: 1.39–3.31).

**Conclusions:**

Plasma proteomics are strong predictors of incident AD/ADRD risk. The identified proteomic markers provide new insights for developing novel risk prediction models, innovative therapeutics, and targeted treatment strategies. The observed racial disparities in AD/ADRD risk warrant further investigation.

